# Risk factors for lymph node metastasis in duodenal neuroendocrine tumors: A retrospective, single-center study

**DOI:** 10.1097/MD.0000000000015885

**Published:** 2019-06-07

**Authors:** Sang Gyu Park, Bong Eun Lee, Gwang Ha Kim, Joon Woo Park, Moon Won Lee, Su Jin Kim, Cheol Woong Choi, Sojeong Lee, Do Youn Park

**Affiliations:** a Department of Internal Medicine, Pusan National University School of Medicine; b Biomedical Research Institute, Pusan National University Hospital, Busan; c Research Institute for Convergence of Biomedical Science and Technology, Pusan National University Yangsan Hospital, Yangsan; d Department of Pathology, Pusan National University School of Medicine, Busan, Republic of Korea.

**Keywords:** duodenal neoplasms, lymph node metastasis, neuroendocrine tumors

## Abstract

Duodenal neuroendocrine tumors (NETs) are rare, and risk factors associated with lymph node (LN) metastasis are still not well defined. The aim of this study was to investigate risk factors of LN metastasis in duodenal NETs based on the final histopathologic results and clinical follow-up data.

This study included a total of 44 duodenal NETs in 38 patients who underwent endoscopic or surgical resection between January 2008 and December 2015. Diagnosis of duodenal NETs was confirmed based on immunohistochemical staining of chromogranin A, synaptophysin, and CD56; the clinicopathologic records were collected at the time of the initial diagnosis of duodenal NETs.

Most duodenal NETs were small (≤1 cm in 33 tumors), World Health Organization (WHO) grade G1 (in 32 tumors), limited to the mucosa and/or submucosa (in 40 tumors), and located at the duodenal bulb (in 32 tumors). Of 44 tumors, lymphovascular invasion was present in 4 (9.1%), and among 38 patients, LN metastasis was detected in 4 (10.5%). LN metastases were significantly associated with the non-bulb location, tumor size >10 mm, tumor invasion into the muscularis propria or deeper, WHO grade G2, and lymphovascular invasion. During the mean follow-up period of 54.5 months (range, 24–123 months), recurrence occurred in 1 patient.

Non-bulb location, tumor size >10 mm, invasion beyond the submucosa, WHO grade G2, and lymphovascular invasion are risk factors of LN metastasis in duodenal NETs. These findings can help clinicians choose the appropriate therapeutic modality for duodenal NETs.

## 1 Introduction

Neuroendocrine neoplasms are defined as neoplasms that arise from cells of the diffuse neuroendocrine system. The World Health Organization (WHO) classified neuroendocrine neoplasms into neuroendocrine tumors (NETs) and neuroendocrine carcinomas according to the tumor grading system in 2010.^[1]^ NETs correspond to neoplasms previously termed as carcinoid tumors. The gastrointestinal (GI) tract is the most frequent site for NETs.^[2]^ Duodenal NETs occur less frequently than gastric and rectal NETs, accounting for <5% of all GI NETs.^[2–4]^ With the widespread use of screening endoscopy, duodenal NETs have been recognized at an increasing frequency. Because GI NETs usually spread to the submucosal layer even at an early stage and the duodenal wall is very thin compared with rest of the GI tract, surgical resection has been accepted as the preferred treatment modality over endoscopic treatment.^[5]^ In a previous study reported in 2003, 13% of duodenal NETs ≤10 mm in size and even 11% of small NETs ≤5 mm in size had lymph node (LN) metastasis.^[6]^

Recently, GI NETs ≤10 mm in size and limited to the submucosal layer are reported to have a low frequency of LN and distant metastases and are good candidates for less invasive treatment modalities, such as endoscopic resection.^[7]^ These less invasive modalities also offer patients an improved quality of life compared with surgical resection.^[2,8]^ As a result, the development of endoscopic techniques has been increasing with reports on endoscopic resection for duodenal NETs ≤10 mm in size.^[9–14]^ In addition, duodenal NETs are usually detected at a very early stage in actual clinical practice.^[15]^ However, because of the rarity of duodenal NETs, there have been few reports on the risk factors of LN metastasis, supporting endoscopic resection for duodenal NETs. Therefore, the aim of this study was to retrospectively investigate the risk factors of LN metastasis in duodenal NETs based on the final histopathologic results and clinical follow-up data.

## 2 Materials and methods

### 2.1 Study population

**Figure 1:**
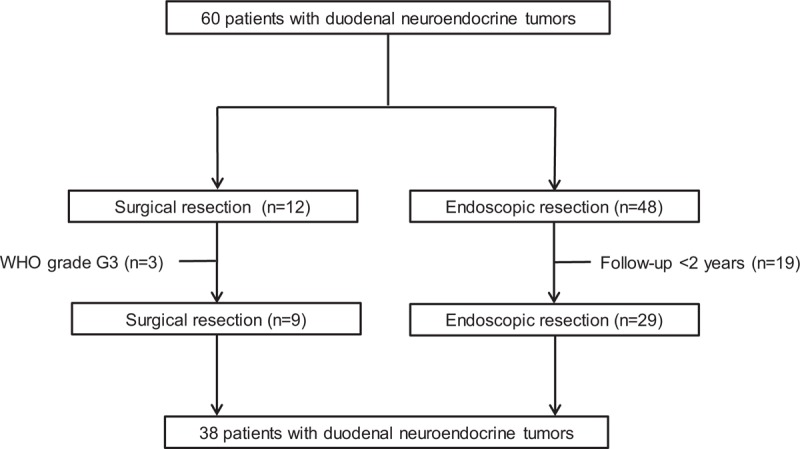
Flowchart showing patient inclusion in this study.

From January 2008 to December 2015, a total of 66 duodenal NETs in 60 consecutive patients who underwent endoscopic or surgical resection were retrospectively enrolled at Pusan National University Hospital (Busan, South Korea) and Pusan National University Yangsan Hospital (Yangsan, South Korea). All lesions were confirmed histopathologically by endoscopic biopsies or resection. Of these, 22 patients were excluded because of follow-up period <24 months (n = 19) and G3 per WHO grading classification (n = 3). Consequently, a total of 44 duodenal NETs in 38 patients were included in the analysis (Fig. 1).

Diagnosis of duodenal NETs was confirmed based on immunohistochemical staining of chromogranin A, synaptophysin, and CD56. The clinicopathologic records were collected at the time of the initial diagnosis of duodenal NETs for information regarding age, sex, tumor location, macroscopic shape, tumor size, presence of multiple endocrine neoplasia type 1 (MEN-1), therapeutic procedure, and presence of LN and distant metastasis. The macroscopic shapes of NETs were classified according to the Paris classification as protruding (I), non-protruding and non-excavated (II), or excavated (III).^[16]^ Type II lesions were subclassified as slightly elevated (IIa), flat (IIb), or slightly depressed (IIc). This study was conducted in accordance with the Declaration of Helsinki, and the study protocol was reviewed and approved by the Institutional Review Board of Pusan National University Hospital (H-1901-025-075).

### 2.2 Histopathological evaluation

Both surgically and endoscopically resected specimens were fixed in formalin, spread out by pins. The surgically resected specimens were opened longitudinally and cut serially into 5-mm thick sections. The endoscopically resected specimens were sectioned serially at 2-mm intervals. The slices were embedded in paraffin, and all sections were stained with hematoxylin and eosin. Immunohistochemical staining of chromogranin A, synaptophysin, and CD56 was performed for confirmation of neuroendocrine differentiation of the tumor. The following histopathologic findings were analyzed: tumor size, depth of invasion, presence of lymphovascular invasion, and WHO grading (Ki-67 index, mitotic index: mitotic count per 10 high-power fields [HPFs]). According to the WHO 2010 grading system, duodenal NETs with <2 mitoses per 10 HPFs, and Ki-67 index ≤2% were regarded as G1, and those with 2 to 20 mitoses per 10 HPFs or Ki-67 index of 3% to 20% were regarded as G2.^[1]^ All resection specimen slides were examined by 2 expert gastrointestinal pathologists (DYP, SJL).

### 2.3 Therapeutic procedure and follow-up

The indications for endoscopic resection were lesions ≤10 mm in size, confined to the submucosal layer as assessed by 12- or 20-MHz catheter probes (UM3D-DP12-25R, UM3D-DP20-25R, Olympus Optical, Tokyo, Japan), without LN or distant metastasis on computed tomography (CT).^[14]^ Otherwise, surgical resection was selected. Endoscopic examinations, with or without biopsies and CT, were performed at 6 and 12 months after resection, and every year thereafter, to check for local recurrence and LN and distant metastasis. Required follow-up duration in cases treated by endoscopic resection in terms of confirming the absence of LN metastasis was assumed to be 24 months at a minimum according to previous studies on duodenal and rectal NETs.^[17,18]^ In cases treated by surgical resection with LN dissection, no metastasis was defined as histopathologically and radiologically proven cases during the follow-up period.

### 2.4 Statistical analysis

Nominal variables are presented as frequencies and percentages, whereas continuous variables are expressed as medians with ranges. The chi-square test and Fisher exact test were performed to identify the risk factors of LN metastasis in duodenal NETs. Statistical calculations were performed using IBM SPSS version 21.0 for Windows (IBM Co., Armonk, NY); results were considered statistically significant when the *P* value was <.05.

## 3 Results

### 3.1 Baseline characteristics of patients with duodenal NETs

The baseline characteristics of the 44 NETs in 38 patients (24 men, 14 women; median age 59 years, range 36–79 years) enrolled in this study are shown in Table 1. Thirty-five patients (92.1%) had no symptoms; almost all duodenal NETs were detected incidentally during screening endoscopy. The remaining 3 patients (7.9%) had symptoms, such as dyspepsia or abdominal pain, and none had MEN-1. Of the 44 tumors, 32 were located in the duodenal bulb, 11 in the second portion of the duodenum, and 1 in the fourth portion of the duodenum. The protruding (type I) and superficial elevated type (type IIa) were the most prevalent types. Most tumors were solitary and ≤20 mm in size. The median tumor size was 0.6 cm (range, 0.2–5.5 cm). Forty tumors were limited to the mucosa and/or submucosa, and the other 4 extended to the muscularis propria or deeper. Lymphovascular invasion was present in 4 tumors (4/44, 9.1%), and LN metastasis was present in 4 patients (4/38, 10.5%).

**Table 1:**
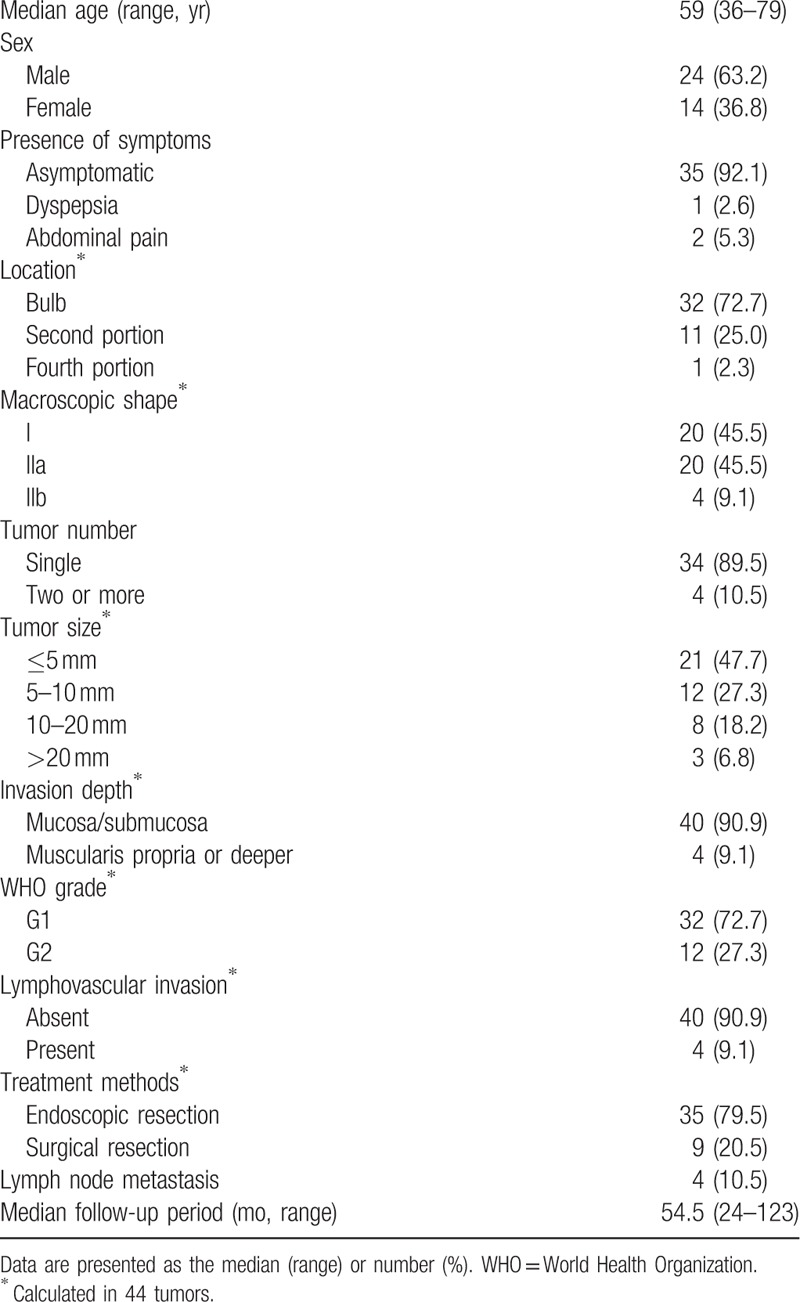
Clinicopathologic characteristics of 44 duodenal neuroendocrine tumors in 38 patients who underwent endoscopic or surgical resection.

Of the 40 tumors limited to the mucosa and/or submucosa, lymphovascular invasion was present in 2 (5.0%); 2 tumors with lymphovascular invasion were 0.6 and 0.7 cm in size, respectively, and had WHO grade G1 and G2, respectively. One patient underwent pylorus-preserving pancreaticoduodenectomy with LN dissection after endoscopic resection, with no evidence of a residual tumor or LN metastasis. The other patient refused additional surgery; neither LN nor distant metastasis occurred during the follow-up period of 49 months. LN metastasis was not observed in any patient with tumors limited to the mucosa and/or submucosa. Of the 4 tumors that extended to the muscularis propria or deeper, lymphovascular invasion was present in 2 (50.0%). LN metastasis was observed in all 4 patients with tumors that extended to the muscularis propria or deeper (Table 2).

**Table 2:**
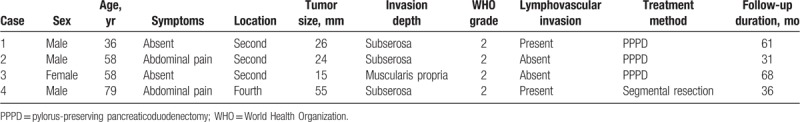
Summary of patients having duodenal neuroendocrine tumors with lymph node metastasis.

### 3.2 Risk factors of LN metastasis in duodenal NETs

In univariate analysis, non-bulb location, tumor size >10 mm, tumor invasion into the muscularis propria or deeper, WHO grade G2, and lymphovascular invasion were significantly associated with LN metastasis (*P* = .004, *P* = .002, *P* < .001, *P* = .004, and *P* = .036, respectively; Table 3). Age, sex, macroscopic shape, and tumor number were not predictive of LN metastasis.

**Table 3:**
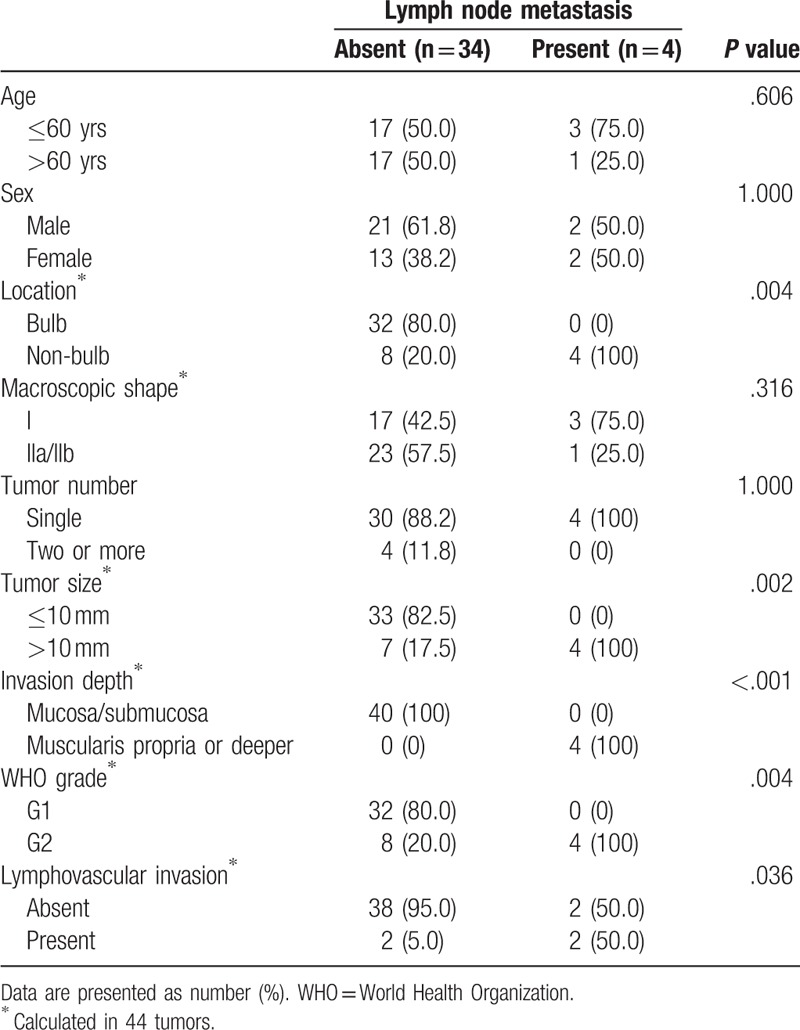
Risk factors for lymph node metastasis in patients with duodenal neuroendocrine tumors.

### 3.3 Follow-up

During the mean follow-up period of 54.5 months (range, 24–123 months), recurrence occurred in 1 patient. He underwent surgical resection for a 5.5-cm-sized NET that extended to the subserosa in the fourth portion of the duodenum, and recurrence occurred in the small bowel mesenteric LN 3 years later. Currently, he has been undergoing chemotherapy. Neither LN nor distant metastasis was observed in the other 37 patients.

## 4 Discussion

For selection of endoscopic resection as the treatment modality for duodenal NETs, the most important issue is the possibility of metastasis. Although a retrospective study reported the absence of recurrence in patients with duodenal NETs <2 cm in size who underwent local excision,^[19]^ another study demonstrated detection of LN metastasis in >10% of patients with duodenal NETs <1 cm in size.^[6]^ Thus, a reasonable consensus has not yet been established regarding the association between tumor size and the possibility of LN metastasis. In the current study, non-bulb location, tumor size >10 mm, invasion beyond the submucosa, WHO grade G2, and lymphovascular invasion were risk factors of LN metastasis in duodenal NETs.

Based on previous reports, duodenal NETs >20 mm in size are considered a risk factor for metastasis.^[7,8,17,19]^ In duodenal NETs ≤20 mm in size, the choice of a treatment modality is still being debated.^[7,11,20]^ The latest European Neuroendocrine Tumor Society guidelines recommend endoscopic resection for duodenal NETs ≤10 mm in size.^[7]^ In the present study, LN metastasis was absent in patients with duodenal NETs ≤10 mm in size, but the LN metastasis rate was 36.4% (4/11) in patients with duodenal NETs >10 mm in size. These results support our previous study; in 33 patients with duodenal NETs ≤10 mm in size confined to the submucosa, there was no recurrence following endoscopic resection during a mean follow-up period of 17 months.^[14]^ However, other studies reported LN metastasis rates of 8% to 13% in duodenal NETs ≤10 mm in size.^[6,17,21]^ In the present study, although LN metastasis was absent in duodenal NETs ≤10 mm in size, lymphovascular invasion, an important risk factor for LN metastasis, was found in 2 tumors (2/33, 6%). Therefore, although tumor size is the main factor for a decision regarding the therapeutic approach to duodenal NETs, detailed histopathologic analysis of resected specimens, and close follow-up are mandatory even after endoscopic resection for duodenal NETs ≤10 mm in size.

In the present study, lymphovascular invasion and WHO grade G2 were significant risk factors for LN metastasis, which is consistent with the results of previous studies.^[17,22,23]^ Although we did not perform multivariate analyses due to the small number of cases, the odds ratio of lymphovascular invasion is reported to be higher than that of the WHO grade 2 (12.5 vs 7.3).^[17]^ Therefore, further research, such as immunohistochemistry with anti D2–40 antibody, to investigate lymphovascular invasion in the resection specimen could be needed. In many studies including the present one, tumor invasion into the muscularis propria or deeper is associated with LN metastasis.^[7,17,24]^ Endoscopic ultrasonography (EUS) is usually used to evaluate the tumor depth and LN involvement in GI NETs.^[24]^ However, there are limitations in the accuracy of EUS in assessing tumor depth; hence, it is mandatory to confirm the final pathological results from resected specimens.

In the present study, the non-bulb location of duodenal NETs was associated with LN metastasis. Of 12 non-bulb NETs, 7 were >1 cm in size and 4 invaded the muscularis propria or deeper. As a result, 7 patients underwent surgical resection. These findings indicate that since the non-bulb portion of the duodenum is a difficult area to observe in detail during screening endoscopy, non-bulb NETs tend to be found later than those located at the bulb. In the present study, we did not measure serum peptide hormone and amine products, such as gastrin, somatostatin, or serotonin. It is reported that almost all duodenal NETs present no functional syndrome despite the positive immunoreactivity for gastrin, somatostatin, and/or serotonin^[25]^ and that only immunoreactivity for gastrin with functional syndrome could be associated with metastasis in duodenal NETs.^[17]^ In the present study, none of the patients had functional syndrome; furthermore, none had MEN-1.

This study has several limitations. First, this was a retrospective study that investigated the risk factors for LN metastasis in duodenal NETs. Therefore, there was a potential bias when retrospectively reviewing the outcomes and selecting the treatment modalities. Second, LN metastasis in the endoscopically resected cases was judged via CT at least 24 months. Considering that CT is not the most appropriate diagnostic modality for LN metastasis, there might be a possibility of underestimation for LN metastasis. In addition, duodenal NETs are slowly progressive tumors, so this period is considered quite short to appropriately confirm the absence of LN metastasis, which could also increase the underestimation for LN metastasis. In fact, the National Comprehensive Cancer Network guidelines recommend follow-up periods of up to 10 years after resection for duodenal NETs.^[1]^ Third, since ampullary NETs have more aggressive clinical features from non-ampullary duodenal NETs,^[7,8,22,26]^ we did not include ampullary NETs in the present study. Finally, the sample size in the present study, especially the number of cases with LN metastasis, was small, which made it very difficult to conduct a multivariate analysis. Therefore, the rarity of duodenal NETs will necessitate multi-institutional studies and larger population-based data sets in order to advance further practices.

## 5 Conclusions

Non-bulb location, tumor size >10 mm, invasion beyond the submucosa, WHO grade G2, and lymphovascular invasion were significant risk factors of LN metastasis in duodenal NETs. These findings can help clinicians choose the appropriate therapeutic modality in duodenal NETs. However, additional multicenter studies involving a larger number of patients with longer follow-up periods are required to validate the above risk factors for LN metastasis in duodenal NETs.

## Author contributions

**Conceptualization:** Gwang Ha Kim, Do Youn Park.

**Data curation:** Sang Kyu Park, Bong Eun Lee, Joon Woo Park, Moon Won Lee.

**Formal analysis:** Su Jin Kim, Cheol Woong Choi, Sojeong Lee.

**Investigation:** Gwang Ha Kim, Do Youn Park.

**Methodology:** Sang Kyu Park, Bong Eun Lee, Cheol Woong Choi.

**Software:** Joon Woo Park, Moon Won Lee, Su Jin Kim.

**Supervision:** Gwang Ha Kim, Do Youn Park.

**Writing – original draft:** Sang Kyu Park, Bong Eun Lee, Gwang Ha Kim.

**Writing – review & editing:** Bong Eun Lee, Gwang Ha Kim.
